# A Developmental Perspective on Underage Alcohol Use

**Published:** 2009

**Authors:** Ann S. Masten, Vivian B. Faden, Robert A. Zucker, Linda P. Spear

**Keywords:** Underage drinking, child, adolescent, alcohol and other drug (AOD) use, abuse, and dependence, AOD use initiation, age of AOD use onset, growth and development, biological development, psychological development, developmental psychopathology, risk and protective factors, genetic factors, environmental factors, AOD effects and consequences

## Abstract

Underage alcohol use can be viewed as a developmental phenomenon because many kinds of developmental changes and expectations appear to influence this behavior and also because it has consequences for development. Data on alcohol use, abuse, and dependence show clear age-related patterns. Moreover, many of the effects that alcohol use has on the drinker, in both the short and long term, depend on the developmental timing of alcohol use or exposure. Finally, many developmental connections have been observed in the risk and protective factors that predict the likelihood of problem alcohol use in young people. Therefore, efforts to understand and address underage drinking would benefit from a developmental perspective, and the general principles of developmental psychopathology offer a useful conceptual framework for research and prevention concerned with underage drinking.

Despite efforts to prevent underage^1^ alcohol consumption, alcohol use remains a pervasive problem among adolescents in the United States. Although the prevalence of underage drinking decreased from its peak in the mid-1970s until about 1993, it has remained relatively constant since that time, with the exception of some recent modest downturns in certain age-groups (Johnston et al. 2007). Underage alcohol use remains at unacceptably high levels across all age-groups. A promising approach to preventing and reducing underage drinking and its adverse effects is to address underage alcohol use as a developmental phenomenon—a problem shaped by the course and contexts of human development and one that also has an array of consequences for development. This article provides a developmental perspective on underage drinking, with a brief overview of the developmental changes of childhood and adolescence, as well as of the interplay of genetic and environmental factors that could play a role in the development and consequences of underage alcohol use. The article then explores various observations supporting the status of alcohol use as a developmental problem, including age-varying patterns of alcohol use and its consequences, alcohol’s effects on development, childhood factors predicting later alcohol use and alcohol use disorders (AUDs), and risk and protective factors associated with alcohol use and dependence. Finally, the article discusses how principles of developmental psychopathology can guide researchers, clinicians, and policymakers in their efforts to understand and address underage drinking.

## A Developmental Perspective

The term “development,” as used in this article, refers to patterns of orderly change that unfold over the lifetime as human beings progress from conception to maturity and then decline and death. Although people change and develop throughout their lives, some of the most rapid and pronounced changes take place during childhood and adolescence. Many of these changes have the potential to affect a young person’s interactions and involvement with alcohol.

### Characterizing Developmental Change

There are several ways to characterize the changes that take place during the first three decades of life. All such descriptions are an attempt to capture the complex, dynamic processes of development from conception to maturity.

One traditional way to describe developmental change is to divide development into age-related segments and delineate normative behaviors and changes that usually occur in these segments of the life course. These developmental categories often begin and end with significant transitions, such as birth or the transition into school. Common developmental categories include the following:

- *Prenatal:* from conception to birth.
- *Early childhood:* from birth to approximately age 5, encompassing infancy, the toddler years, and the preschool period.
- *Middle childhood:* from entering school (around age 4 or 5) to the transition into adolescence, which is heralded by signs of puberty and changes in school or social contexts (around age 8 to 10).
- *Adolescence:* encompasses the onset of puberty, secondary school transitions, and the second decade of life (from around age 8 to 10 to approximately age 18 to 20).
- *Transition-to-adulthood* (sometimes called “emerging adulthood”): from approximately age 18 to 25.

A second way to describe developmental change is in terms of developmental tasks and accomplishments characteristically expected and achieved during a given time period. Some of these tasks are universal, whereas others are specific to a given culture, place, or time in history (for more information, see also the textboxes “Developmental Tasks and Transitions” and “Appropriate Drinking Behavior as a Developmental Task”). Examples of common developmental tasks in many contemporary societies include the following:

**In early childhood:**
- Forming attachment bonds with caregivers;
- Talking and learning the native language of the family; and
- Complying with and following simple adult commands.

**In middle childhood:**
- Adjusting to school;
- Learning to count, read, and do basic mathematics;
- Getting along with peers at school and making friends; and
- Engaging in rule-abiding behavior at home and school.

**In adolescence:**
- Achieving academic success in more advanced topics;
- Graduating from high school;
- Making and maintaining close friends; and
- Learning and following the rules and laws that govern conduct in society.

**In early adulthood:**
- Achieving higher education or vocational training;
- Gaining employment or other kinds of work;
- Forming a romantic or marital partnership;
- Engaging in responsible sexual behavior; and
- Parenting effectively when one becomes a parent.

**Developmental Tasks and Transitions**

Parents and societies throughout the world develop expectations and standards as to what children should be doing at various maturational stages as they progress toward adulthood and successful family and social roles (Masten et al. 2006). These expectations, which children themselves come to share or, perhaps, rebel against, include a wide range of behaviors. Examples are following the rules and laws that govern conduct in adult society, engaging in responsible dating or romantic social conduct, learning to drive a car, contributing to the family or community through chores or good deeds, and joining a religious community. Failing to meet developmental expectations may have serious consequences regarding a child’s current and future opportunities, reputation among peers, social support, self-esteem, and parent–child relationships. Some of these expectations are universal (e.g., learning to talk), whereas others are highly specific to a culture or region. Across all cultures, important transitions have traditionally been celebrated, facilitated, and supported through familial, social, and religious rituals, ceremonies, specialized activities, or structured experiences.

**Appropriate Drinking Behavior as a Developmental Task**

In societies where alcohol use is pervasive and a widely accepted behavior for adults, it could be argued that developing an appropriate relationship with alcohol (whether abstinence or socially appropriate use) itself is an important developmental task (Masten et al. 2008). Parents have a key role in helping their children develop the skills to achieve responsible adult use or abstinence.

Developmental changes can also be categorized in terms of changes in developmental processes at multiple levels of functioning, as reflected in changing adaptive capacities of the person. With this approach, developmental processes often are described in relation to changes in biological processes (e.g., brain development, puberty, growth), cognitive processes (e.g., perception, memory, executive functioning), and social–emotional processes (e.g., personality, motivation, relationships).

A fourth way to describe change focuses on the changing contexts in which an individual lives and interacts. Some changes are arranged by societal or community practices, such as school changes, whereas others result from life events, such as moving or disasters. Contextual changes bring new challenges and opportunities; alter the nature of parental, school, and other types of support; and increase life’s complexity. The extent of adult monitoring changes with age, developmental progress, and contexts. The primary contexts that shift are:

- *Physical contexts—* for example, the places where children spend their time, such as at home, at school, on the playground, in extracurricular activities, in the mall, or in other gathering places;
- *Social contexts*—for example, the network of people and social situations within which children interact on a routine basis, such as family, friends, classmates, sport teams, parties, etc.;
- *Societal and cultural contexts*—for example, ethnic, community, civic, and religious activities, belief systems, expectations, and rituals; military service;
- *Media and virtual environments*—for example, movies, television, the Internet, computer games, e-mail, instant messaging, and magazines, often resulting in exposure to adult content.

Finally, it is possible to describe changes taking place in the interactions of individuals with other people and other contexts as all this change is occurring.

Collectively, these different ways to describe the nature of change in human development are attempts to simplify, yet capture, the highly complex processes of human maturation and the various influences involved. Although development is related to age, it is not the same thing as aging, which is one reason why it is difficult to “slice” development neatly by age. The timing of developmental changes varies, both within and across individuals. Some children begin the growth spurt of puberty earlier or later than others and grow more quickly or more slowly than others. Moreover, the growth spurt typically begins and peaks at younger ages for most girls compared with most boys. The result of this variable development can readily be observed in a group of young adolescents who are all the same age but clearly differ in pubertal development as well as height. Even within the same child, different aspects of development may have different timing. For example, a child may begin to walk earlier than most children but begin to talk later than most.

These multiple, simultaneously occurring developmental processes, combined with variations in timing and tempo, can result in interesting gaps between the maturity of development in one area of function compared with another. Just as a toddler may learn to walk before his or her judgment to stay out of the street is in place, a teenager may become motivated for adventure or romantic encounters before mature decisionmaking and planning are fully developed. For example, within the developing brain, the regions governing some emotional and motivational systems mature early in adolescence (linked to the onset of puberty), whereas the systems responsible for considering future consequences as well as for cognitive and self-regulatory controls mature more gradually throughout adolescence and into early adulthood, creating a developmental dysynchrony that may help explain the increase in risk-taking behavior in adolescence (Dahl 2004; Romer and Walker 2007; Steinberg et al. 2006). Such gaps may be more pronounced in some individuals, depending on the patterning of their development in various areas of function.

Over the past 100 years, the age of onset of puberty and sexual maturation has decreased, most likely as a result of changes in diet and health. At the same time, young people in industrialized societies require more time than preceding generations to become established as adults in work and family life. This trend, which likely results at least in part from increased training and education requirements, has extended the time period of adolescence. In many modern societies, an extended transition period—recently described as “emerging adulthood” (Arnett 2000)—now exists between adolescence and full adult status that lacks the constraints imposed by the obligations of full adulthood.

### The Interplay of Genes and Environment in Development

At one time, it was thought that genes and their function were static, were impervious to the effects of life experience, and could be described as a “blueprint” for building development. At that time, much discussion focused on “nature” (i.e., genes) versus “nurture” (i.e., environment) as explanatory causes of human behavior and outcomes. That notion now has been replaced by the realization that genes and environment do not influence development independently but rather are inextricably bound through complex interactions involving bidirectional influences of genes and environments. The term “epigenesis” captures this idea of development unfolding from the interplay of genes and environment through many interactions over time. In its broadest sense, epigenesis refers to the dynamic, complex processes by which genes respond to each other and to environmental signals throughout a lifetime to produce an individual who is adaptive and functional (Gottesman and Hanson 2005). Interactions between genes, neurons, behavior, and contexts explain why the same genes can result in very different outcomes, depending on when and where specific genes are activated or repressed. For example, these complex interactions between genes and the environment explain why identical twins differentiate over the course of their development.

Although no definitive answers are as yet available, alcohol researchers are particularly interested in two areas of gene–environment interactions. The first area is the identification of specific genes that might interact with experience during the course of human development to increase or decrease the likelihood of alcohol use and AUDs. A second area of interest is the effect of ethanol exposure on gene expression in various tissues and organs, particularly the brain and liver, across human development.

### Scaffolding for Positive Development

Across the millennia, parents have learned that certain developmental transitions can be hazardous, reflecting gaps in maturity between capacities and desires. Toddlers who have just learned to walk and adolescents who have just learned to drive share a common elevated risk for accidents related to a mismatch between their new capability and the judgment and experience required to handle it safely. In addition, there are periods of concentrated change in individuals and their contexts that create windows of both opportunity and vulnerability for the developing individual (Dahl 2004; Masten 2004, 2007*b*; Steinberg et al. 2006).

One of the most important roles adults and society have is to protect children and youth from harm, especially during periods of increased risk, by guiding, monitoring, disciplining, and supporting them in ways that are appropriate to their level of maturity, so that they can function beyond their independent capabilities or despite their vulnerabilities as they move toward adulthood (Masten 2004, 2007*b*). The broad term for this function in developmental science is scaffolding (Vygotsky 1978). A good example of a social scaffolding is a graduated driver’s license, which attempts to reduce the risks to beginning adolescent drivers by setting limits on where, when, and with whom they can drive.

## Underage Alcohol Use as a Developmental Phenomenon

Each of the following observations supports the premise that underage alcohol use is a developmental phenomenon:

- Alcohol use, problems, abuse, and dependence have striking age-related patterns.
- The acute, intermediate, and longer-term effects of alcohol vary by age and development.
- Development itself may be altered by alcohol exposure.
- The likelihood of an adolescent eventually using alcohol or manifesting AUDs can be predicted from childhood factors.
- Risk and protective factors associated with higher or lower use/dependence have age-related patterns.

The following sections provide evidence for each of these observations.

### Alcohol Use, Problems, Abuse, and Dependence Have Striking Age-Related Patterns

**Figure 1:**
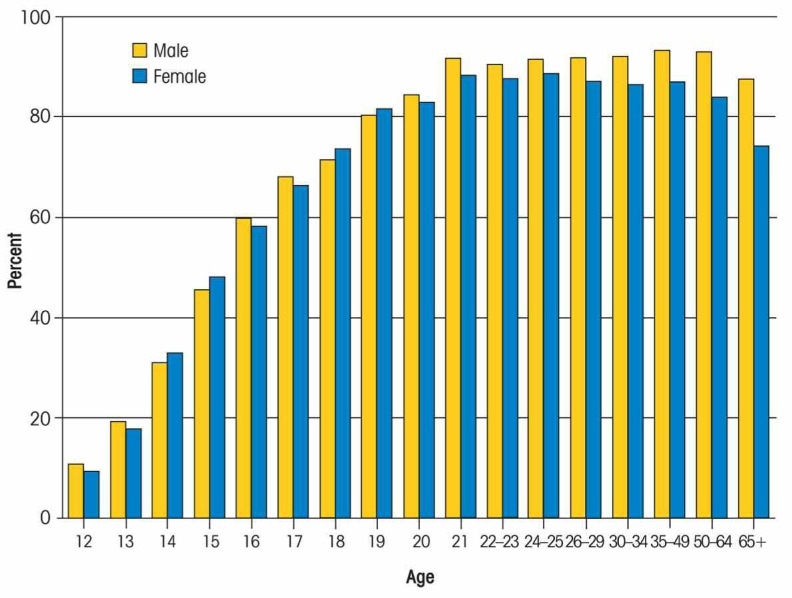
Percentage of americans who have ever drunk alcohol (i.e., consumed a whole drink). SOURCE: SAMHSA, National Survey on Drug Use and Health, 2007.

**Figure 2:**
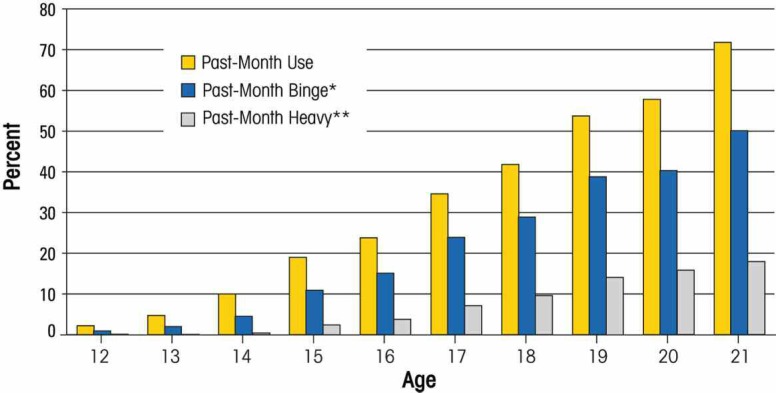
Past-month alcohol use (any, binge, heavy) by age. NOTE: *Binge is defined as five or more drinks on an occasion. **Heavy Drinking is defined as five or more drinks on an occasion on five or more of the past 30 days. SOURCE: SAMHSA, National Survey on Drug Use and Health, 2007.

Alcohol use, problems, abuse, and dependence are related to age in multiple ways. Although some children begin drinking in elementary school, alcohol use (defined as drinking a whole drink) typically begins in early adolescence, around ages 12–14 (Faden 2006). Between ages 12 and 21, rates of alcohol use and binge alcohol use increase sharply. For example, data from the 2005 National Survey on Drug Use and Health (NSDUH) indicate that the proportion of youth who have ever drunk alcohol rises steeply during adolescence, leveling off around age 21 (see figure 1). Data from the same study (see figure 2) indicate that all levels of past-month alcohol usage increase steadily from ages 12 to 21, including any alcohol use (defined as drinking at least one whole drink in the past month), binge use (defined as drinking four or more drinks on one occasion), and heavy use (defined as drinking five or more drinks on five or more days within the past month).

**Figure 3:**
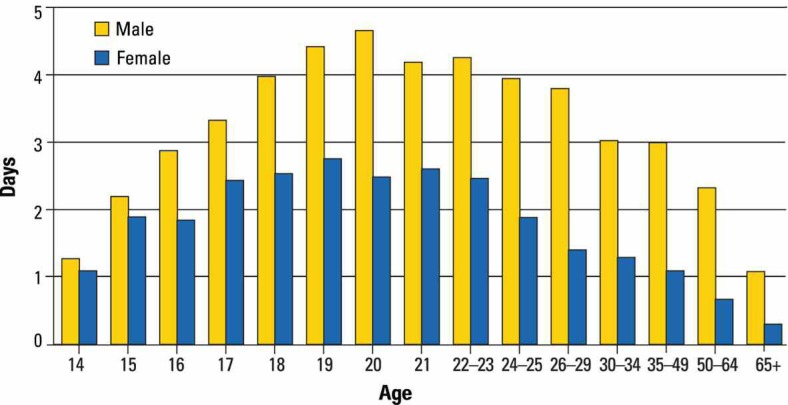
Number of days in the past 30 days on which drinkers consumed five or more drinks, by age and gender. SOURCE: SAMHSA, National Survey on Drug Use and Health, 2007.

The number of drinking days in the past 30 days on which youth engaged in binge-drinking behavior shows another age-related pattern. Binge drinking increases sharply during adolescence. As indicated in figure 3, the number of binge-drinking days in the past 30 days increased continuously for males from age 14 to age 20; for females, the number of binge-drinking days also rose during that time, although less dramatically. During the third decade of life and thereafter, the number of binge-drinking days in the past 30 days declined for both sexes.

**Figure 4:**
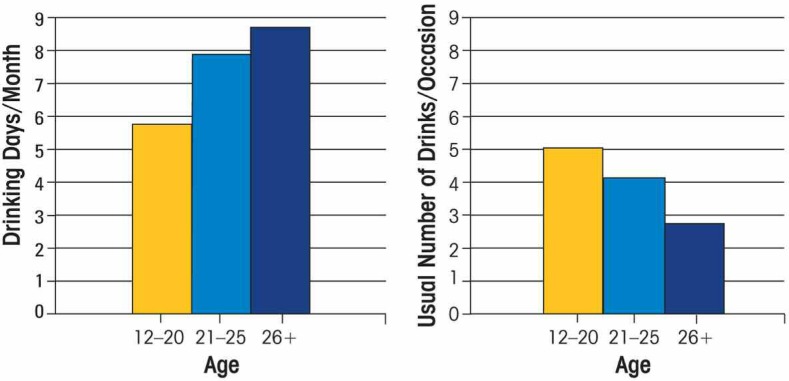
Number of drinking days per month and usual number of drinks per occasion for youth (ages 12 to 20), young adults (ages 21 to 25), and adults (ages 26 and older). SOURCE: SAMHSA, National Survey on Drug Use and Health, 2007.

The NSDUH data also indicate that adolescents drink less often than adults but drink more than adults per drinking occasion (see figure 4). When young people drink, they consume on average about five drinks, which constitutes binge drinking (typically defined as consuming five or more drinks per occasion for men and four or more per occasion for women). Certain youth-oriented settings that attract adolescents, such as teen parties, college, and military service, are associated with high rates of drinking, especially binge drinking behavior (Bray et al. 2006; National Institute on Alcohol Abuse and Alcoholism [NIAAA] 2002). In fact, underage drinking accounts for a substantial portion of all alcohol consumed in the United States and, therefore, of consumer expenditures for alcohol. The estimated short-term cash value to the alcohol industry of underage drinking was $22.5 billion in 2001(Foster et al. 2006).

**Figure 5:**
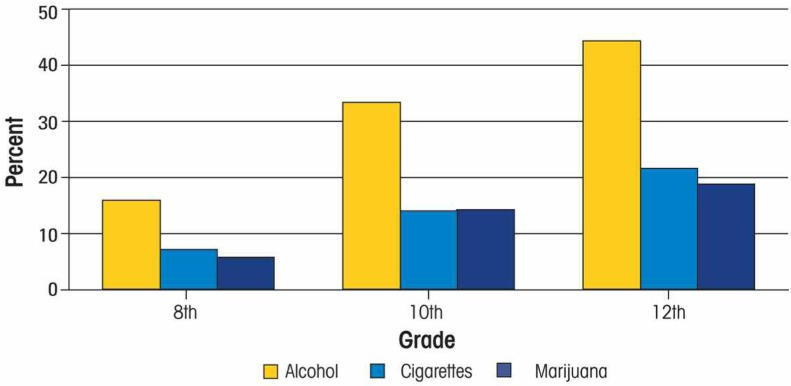
Past-month adolescent alcohol, cigarette, and marijuana use by grade according to the 2007 monitoring the future survey. SOURCE: Johnston et al. 2007.

Various nationally representative surveys confirm that alcohol is the drug of choice among American adolescents of all ages. Data from the Monitoring the Future Survey indicate that more 8th-, 10th-, and 12th-grade youth drink alcohol than smoke cigarettes or use marijuana (see figure 5) (Johnston et al. 2006). The percentages for males are even more dramatic: 50.7 percent of 12th-grade males reported having consumed alcohol in the past month.

**Figure 6:**
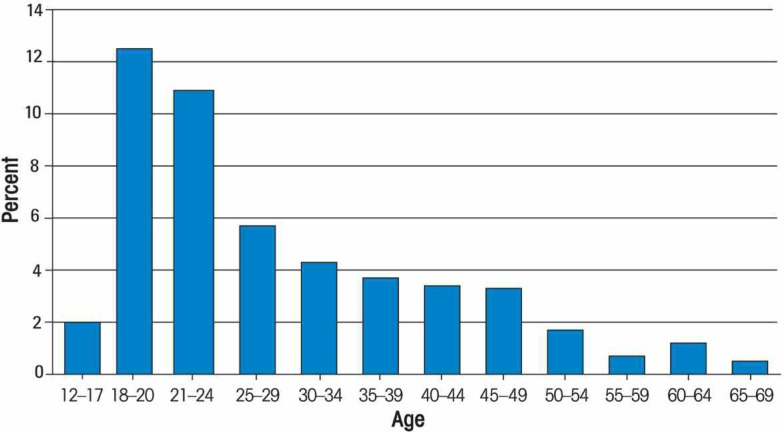
Prevalence of past-year dsm–iv alcohol dependence in the united states. SOURCE: 18+ years: 2001–2002 National Epidemiologic Survey on Alcohol and Related Conditions, 12–17 years: National Survey on Drug Use and Health, 2003.

**Figure 7:**
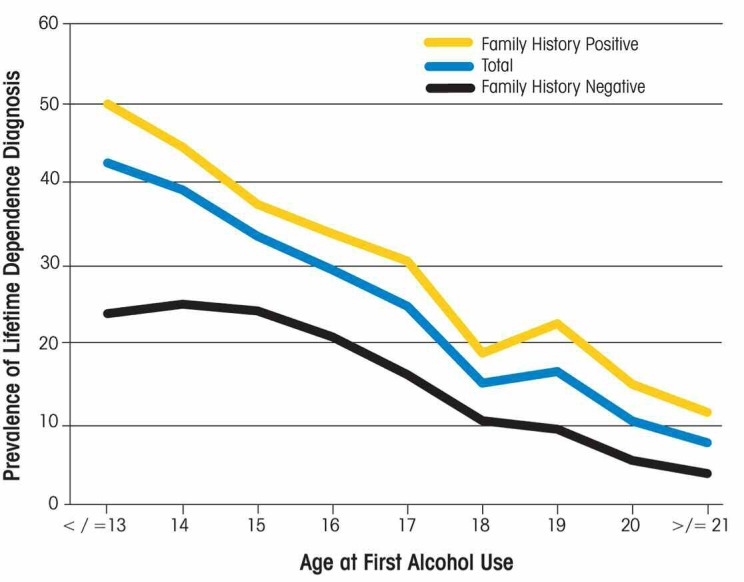
Association between age at initiation of alcohol use and lifetime dependence (i.e., meeting the DSM–IV criteria for dependence at some point in life). The blue curve represents all respondents, the yellow curve represents respondents with a family history of alcoholism, and the black curve represents respondents without a family history of alcoholism. SOURCE: 2001–2002 National Epidemiologic Survey on Alcohol and Related Conditions.

Alcohol dependence, as defined by the criteria of the American Psychiatric Association in the *Diagnostic and Statistical Manual of Mental Disorders* (DSM–IV), typically emerges during late adolescence or early in the young-adult years. As shown in figure 6, past-year prevalence of DSM–IV alcohol dependence dramatically increases between ages 12 and 20 and peaks between ages 18 and 20. Moreover, children and youth whose alcohol use begins earlier (typically in childhood or early adolescence) are much more likely to develop alcohol dependence (see figure 7).

### Acute, Intermediate, and Longer-Term Effects of Alcohol Exposure Vary by Age and Development

Research data from animal research and a limited number of human studies indicate that the acute (i.e., immediate), short-term, and long-term effects of alcohol on individuals vary in part as a function of the age or development of that individual. (For information on the use of animal models of development, particularly in research on underage drinking, see the sidebar “Animal Models of Development: What They Tell Us.”) For example, prenatal versus postnatal exposure to alcohol has dramatically different consequences for development in both humans (Brown and Tapert 2004; Jones and Smith 1973; White and Swartzwelder 2004) and animals (Sulik et al. 1981). Moreover, fetal alcohol spectrum disorders (FASD), which result from alcohol consumption during pregnancy, create a wide range of anomalies in humans and animals. In rhesus monkeys, different effects on fetal development result from differences in the timing of prenatal exposure to alcohol (Schneider et al. 2005). Animal research also indicates that alcohol consumption prior to and during adolescence can produce long-lasting effects in the organism, including increased alcohol consumption in adulthood (McBride et al. 2005).

Data from animal research strongly suggest that compared with adults, adolescent animals are less sensitive to the negative effects of acute alcohol intoxication such as sedation, hangover, and loss of muscular coordination but are more sensitive to alcohol’s effects on social facilitation and disruption of spatial memory (Spear and Varlinskaya 2005; also see the article by Windle and colleagues, pp. 30–40 in this issue). Similarly, human adolescents may be less sensitive than adults to particular effects of alcohol and therefore may be at higher risk for heavier consumption per drinking occasion.

An association also has been demonstrated between alcohol consumption and stress. For example, monkeys that were raised by peers (an animal model of early stress) as adolescents increase their alcohol intake under stressful conditions substantially more than do adolescent monkeys which were raised in a nonstressful environment by their mothers. In addition, excessive alcohol consumption is related to changes in stress hormones and the brain signaling molecule (i.e., neuro-transmitter) serotonin (Barr et al. 2004).

Research on the long-term consequences of chronic alcohol exposure in animals also suggests differences between adolescents and adults in their vulnerability to adverse effects on the brain. In one study (White et al. 2000), rats were exposed to chronic, intermittent alcohol exposure as adolescents or early adults. After a 20-day recovery period, learning was significantly more impaired in the animals that were exposed to alcohol in adolescence than in the animals exposed as adults when both groups of animals were challenged with a low dose of alcohol. A study in which a very high dose of alcohol was applied to adolescent and adult rats over a 4-day period found that certain brain regions may be more susceptible to alcohol-induced damage during adolescence than during adulthood (Crews et al. 2000). Studies involving human adolescents in treatment for AUDs indicate that severe AUDs are associated with reduced volume of a brain region called the hippocampus (DeBellis et al. 2000; Nagel et al. 2005), although more studies are needed to explore causality.

### Development Itself May Be Altered By Alcohol Exposure

Exposure to alcohol during fetal development, childhood, and adolescence can alter development itself. The developing embryo and fetus are particularly vulnerable to the adverse effects of alcohol. Alcohol-induced birth defects are known as FASD. The most serious of these is fetal alcohol syndrome (FAS), a developmental disorder characterized by abnormalities in the head shape and face (i.e., craniofacial abnormalities), growth retardation, and nervous system impairments that may include mental retardation. Alcohol also may damage neurological and behavioral development even in the absence of obvious physical birth defects. The severity of defects depends on the dose, pattern, and timing of in utero exposure to alcohol.

Alcohol use by children and adolescents also may impair their development. During this period, individuals are developing social and academic competencies that are critical to becoming a successful adult (National Research Council and Institute of Medicine 2004; Zucker 2006). Drinking contributes to problems in key behavioral domains of children and adolescents, such as peer relationships and school performance. For example, underage drinking can interfere with school attendance, disrupt concentration, damage relationships, and potentially alter brain function and/or other aspects of development, all of which have consequences for future success in such areas as work, adult relationships, health, and well-being. In other words, developmental cascades, or “snowball” effects can occur in which alcohol affects one aspect of development, leading to other problems in the course of development (Masten et al. 2005).

In addition to drinking by youth themselves, alcohol use by parents, teachers, and other adults who play a key role in a child’s development also can undermine the achievement of developmental tasks. For example, inappropriate adult alcohol use can result in prenatal or postnatal exposure to alcohol; interfere with parenting; contribute to poverty, neglect, and family violence; increase the risk of a child’s involvement with deviant peers; and in other ways increase the general level of risk and adversity faced by a child.

### Childhood Factors Predict Future Alcohol Use and Alcohol Use Disorders

Substantial research has implicated a set of risk factors that consistently precede and predict early alcohol use and/or dependence (National Research Council and Institute of Medicine 2004; Zucker 2006; Donovan 2004; NIAAA 2004–2005). These alcohol-specific risk factors include the following:

- Prenatal exposure to alcohol, including that which gives rise to FASD, including FAS;
- A family history of alcohol abuse, antisocial behavior (by either parent), and depression (in the mother);
- Poor parenting of the child (e.g., maltreatment, neglect, poor monitoring);
- Childhood antisocial behavior;
- Childhood smoking or other kinds of substance use;
- Early signs of cognitive and learning problems, including academic failures; and
- Self-regulation problems that also predict antisocial and risk-taking behavior, such as attention problems, difficulty regulating emotion or behavior, poor impulse control, and effortful control problems.^2^

A majority of the risk factors for alcohol use and AUDs, however, are nonspecific to alcohol involvement—that is, they also predict many other kinds of problems, such as conduct and learning problems, risk-taking behaviors, dropping out of school, early sexual activity and pregnancy, antisocial personality disorder, and mood disorder (Dodge and Pettit 2003; Evans et al. 2005; Kendler et al. 2003; NIAAA 2004–2005; Tsuang et al. 1998; Zucker 2006). Many of these nonspecific risk factors already are evident in preschool age children, including the following:

- Temperament differences related to behavioral and emotional control;
- Problems with self-awareness and self-monitoring, attention, response inhibition, and effortful control; and
- A history of adversity in multiple forms, such as a family history of antisocial behavior, abuse and trauma, or other negative life experiences.

### Risk and Protective Factors Associated With Higher or Lower Use/Dependence Have Age-Related Patterns

Of particular interest to parents and society are factors that either increase the risk of alcohol use/dependence or decrease that risk. Research indicates that both risk and protective factors have age-related patterns. For example:

- The intent to use alcohol increases with age during elementary school (Donovan et al. 2004).
- Expectations about the effects of alcohol use shift from predominantly negative to positive during late middle childhood and early adolescence ([Bibr b27-arh-32-1-3][Bibr b28-arh-32-1-3]). These changes may be linked to the transition from childhood to adolescence or from elementary school to secondary school. An analysis of the Pittsburgh Girls Study, for example, indicates that positive expectations about alcohol use rose and negative expectations fell between ages 8 and 10 (Hipwell et al. 2005). Dunn and Goldman (1996, 1998) also found that the shift in expectations occurs earlier than the transition from elementary school to middle school. (For more information on the role of middle childhood in the development of alcohol use behaviors, see the textbox “Middle Childhood.”)
- Access to alcohol tends to increase over the course of childhood and adolescence (Johnston et al. 2006).
- Popularity with peers generally is associated with lower risk for alcohol use in elementary school (Zucker 2006), but popular high school students may a have higher risk (Diego et al. 2003). Some of that heightened risk may result from the increased exposure to alcohol at parties that occurs with adolescence, because popular youth are more likely to be invited to parties.
- The timing of physical maturation has significant ramifications for social interactions and alcohol use. Early-maturing girls, for example, may date older boys who drink at parties and may find themselves unable to deal with resulting situations.
- Underage drinking is viewed as an adolescent rite of passage by many American parents and also by many adolescents (Jessor and Jessor 1977; Maddox and McCall 1964). Childhood drinking, on the other hand, generally is not culturally acceptable; therefore, a shift in adult expectations about adolescent alcohol use and at least tacit approval of drinking must also occur.
- The transition to college significantly increases the risk for binge drinking, particularly in the first few months of the freshman year (White et al. 2006). A subset of college binge drinkers already have been drinking at high levels in high school and continue this practice in college. Another group increases their alcohol consumption at the beginning of college but then reduces it. For still others, the risk of binge drinking declines (Schulenberg et al. 1996; also see the article by Brown and colleagues, pp. 41–52 in this issue).
- Smoking, which is a risk factor for alcohol use, typically begins in early adolescence (Klein 2006).
- Associating with deviant peers and delinquent behaviors among deviant peers both are key risk factors for alcohol use. They increase in early adolescence, especially among youth characterized by a cluster of risk factors for antisocial and risk-taking behavior (Dishion and Patterson 2006).
- A decline in parental and other adult monitoring, which can be protective, often occurs during adolescence, and unmonitored adolescent time increases, which can heighten risk.

## Using Principles of Developmental Psychopathology to Understand and Address Underage Drinking

The core assumptions and guiding principles of developmental psychopathology provide a useful conceptual framework for understanding and addressing behavioral problems and disorders, including underage drinking, in relation to developmental issues (Cicchetti and Cohen 2006; Cummings et al. 2000; Masten 2006). The major principles of developmental psychopathology and how they may relate to underage alcohol use are briefly described below.

### The Developmental Principle

People develop and change across the lifespan, and therefore a developmental perspective is necessary to understand, prevent, and treat the causes and consequences of behavioral problems.

Many influences and interactions involving the complex interplay of genes and environment shape the course of human development (Gottesman and Hanson 2005; Masten 2007*a*). As a result, individual development can take many directions. Multiple causes can contribute to a given problem or disorder, and there can be a multiplicity of consequences. Thus, there are multiple pathways leading toward the same problem and multiple pathways leading away from it. With respect to alcohol use, problems can arise in an individual with a normal childhood and few risk factors as well as in one whose development was troubled all along the way by many risk factors, family dysfunction, and early or ongoing mental health problems. On the other hand, children who share very similar or identical risk factors for alcohol use may follow very different paths through life, some developing and others completely avoiding alcohol-related problems. The “causes” of developing an AUD (as well as the causes of avoiding an AUD) in these contrasting developmental patterns are likely to differ.

Human development generally involves periods of continuity and orderly change as well as intervals of discontinuity and transformation. Rapid change and transformation create instability and hence offer windows of vulnerability as well as opportunities for shifting development to a more positive developmental course. Interventions that incorporate a developmental perspective attempt to interrupt negative developmental processes or take advantage of opportunities for utilizing developmentally relevant leverage. They also take into consideration individual differences in their timing and design as well as address multiple levels of risk and protective factors. For example, an intervention might take advantage of developmental periods when approachability is high and risk levels are relatively low (e.g., middle childhood) or leverage developmental factors that contribute to change, such as peer influence (Masten et al. 2006; Zucker and Wong 2005; see also the article by Wagner, pp. 67–75 in this issue).

### The Normative/Expected Principle

Behavioral problems and disorders are defined in relation to development that is considered normative or expected by a given culture or society at a given time in history.

Every society has a set of shared assumptions about what constitutes normal human development, based in part on what has been observed to be typical of development across the lifespan and additionally influenced by cultural norms. These assumptions generate expectations about behavior that vary by age, society, and subgroups within societies; moreover, these expectations may change as societies change. Developmental tasks reflect these normative expectations, as described above. However, just because a behavior is normative or typical (i.e., manifested by the majority of people in a society) does not mean that it is considered acceptable. It is possible for a behavior to be normative and unacceptable. In the case of alcohol use, there are three possible combinations: (1) normative and acceptable (e.g., a 21-year-old having a drink), (2) normative and unacceptable (e.g., a 17-year-old having a drink), and (3) nonnormative and unacceptable (e.g., a 6-year-old having a drink, which is neither normative nor acceptable in most societies).

**Animal Models of Development: What They Tell Us**

Animal models play an important role in alcohol research because it is unethical to conduct genetic experiments on humans or to give alcohol to youth to determine the short-and long-term effects of alcohol on their developing bodies and minds. Yet such experiments are important for understanding underage alcohol use as a developmental phenomenon and the effects of alcohol on human maturation. Therefore, researchers turn to animals, in which direct experimentation is permitted. Fortunately, other mammals, like humans, undergo an adolescent period in which they mature sexually and develop the skills to survive away from their parental caretakers (Spear 2000). A broad range of species from rodents to primates share similarities with human adolescents in their fundamental neural, hormonal, and behavioral characteristics (Dahl and Spear 2004; Romer and Walker 2007; Spear 2000). For example, significant similarities exist between adolescent humans and other mammalian species in brain sculpting (Spear 2000) as well as in characteristic adolescent behaviors, such as increased risk-taking, sensation- or novelty-seeking (Douglas et al. 2003; Trimpop et al. 1999), and focus on social interactions with peers (Csikszentmihalyi et al. 1977; Douglas et al. 2004). An example of risk-taking in human adolescents (Johnston et al. 2006) as well as in adolescents of other species (Doremus et al. 2005) is the propensity to drink substantial amounts of alcohol despite various adverse consequences (McBride et al. 2005; Windle and Windle 2005).

Although animal models can provide important information in certain areas, many critical aspects of human development cannot be studied with animal models because of the unique complexity of the human brain and of human behavior and cognition. For example, the impact of advertising on alcohol consumption, the effect of laws against underage drinking, and ethnic differences in acceptability of alcohol use are not suitable for study through animal models. Hence, animal models must be used judiciously, depending on the aspect of human development being studied. Nonetheless, in some areas animal models can contribute important data. Examples include the long-lasting consequences of early alcohol exposure on neurocognitive function and behavior, or how interactions among brain, behavior, and environment lead to excessive adolescent alcohol consumption.

ReferencesCsikszentmihalyiMLarsonRPrescottSThe ecology of adolescent activity and experienceJournal of Youth and Adolescence6281294197710.1007/BF0213894024408457DahlRESpearLPAdolescent Brain Development: Vulnerabilities and Opportunities: Annals of the New York Academy of Sciences, 1021Annals of the New York Academy of Sciences; No. 1021200410.1196/annals.1308.00115251869DoremusTLBrunellSCRajendranPSpearLPFactors influencing elevated ethanol consumption in adolescent relative to adult ratsAlcoholism: Clinical and Experimental Research29101796180820051626990910.1097/01.alc.0000183007.65998.aaDouglasLAVarlinskayaEISpearLPNovel-object place conditioning in adolescent and adult male and female rats: Effects of social isolationPhysiology & Behavior8031732520031463723110.1016/j.physbeh.2003.08.003DouglasLAVarlinskayaEISpearLPRewarding properties of social interactions in adolescent and adult male and female rats: Impact of social versus isolate housing of subjects and partnersDevelopmental Psychobiology4515316220041550579710.1002/dev.20025JohnstonLDO’MalleyPMBachmanJGMonitoring the Future, National Survey Results on Drug Use, 1975–2005. Volume 1: Secondary School StudentsNIH Pub. No. 06–5883Bethesda, MDNational Institute on Drug Abuse2006McBrideWJBellRLRoddZAAdolescent alcohol drinking and its long-range consequences: Studies with animal modelsRecent Developments in Alcoholism1712314220051578986310.1007/0-306-48626-1_6RomerDWalkerEFAdolescent Psychopathology and the Developing BrainNew YorkOxford University Press2007SpearLPThe adolescent brain and age-related behavioral manifestationsNeuroscience and Biobehavioral Reviews24441746320001081784310.1016/s0149-7634(00)00014-2TrimpopRMKerrJHKirkcaldyBComparing personality constructs of risk-taking behaviorPersonality and Individual Differences2622372541999WindleMWindleRCAlcohol consumption and its consequences among adolescents and young adultsRecent Developments in Alcoholism17678320051578986010.1007/0-306-48626-1_4

**Middle Childhood**

Some brain functions, such as those associated with self-regulation and decisionmaking, develop more gradually than others and primarily are a function of age and experience. This developmental lag time, coupled with the increasingly earlier onset of puberty, has narrowed the developmental period that used to be called “middle childhood” or “latency” between the beginning of school and the onset of puberty. These younger pubescent children attend elementary school but have the interests of adolescents. As so-called “tweens,” they have been targeted by marketers and the media with the kind of specialized clothing, Web sites, movies, and other products (Brown and Witherspoon 2002; Steinberg et al. 2006) more traditionally associated with older adolescents. As tweens take on the attitudes and behaviors of their older adolescent peers, there is growing concern that one of the new interests they will share is alcohol (Donovan 2004). As young adolescents mature earlier and encounter greater risks around alcohol use, they need adequate monitoring and support to counter those risk factors that have heightened with increased media exposure, disrupted families, and increased alcohol use among deviant peers (Steinberg et al. 2006; also see the article by Windle and colleagues, pp. 30–40 in this issue).

### The Systems Principle

Human beings are living systems and, therefore, behavior problems and disorders emerge from complex interactions among systems within individuals and also between an individual and the multiple systems that affect his or her life.

Underage drinking results from the interactions of many systems within an individual (e.g., the central nervous system) as well as between the individual and other external systems (e.g., family, peers, school, media, religion, and cultural group). Genes, individual motives and desires, family functioning, community values, media messages, friendships, romantic partners, school norms, religious beliefs, adult monitoring, the price and availability of alcohol, and many other factors collectively determine adolescent behavior in connection with alcohol. Furthermore, the relative importance of each of these factors varies as development proceeds. For example, peer influence becomes increasingly important during late childhood and early adolescence.

### The Multilevel Principle

Interactions across multiple levels of function (molecular to societal) are involved in the processes that shape development, and therefore it is important to consider multilevel processes for understanding pathways to problems and interventions to prevent or address problems or disorders in development.

A corollary of the systems principle, the multilevel principle recognizes the potential role of multiple levels of processes in the development of psychopathology. Understanding the origins of drinking and the possible strategies for addressing underage drinking from a multiple-levels perspective requires the contributions of multiple disciplines. Over the course of development, the costs and benefits for interventions targeting particular levels or systems may vary. For example, as the influence of peers or the social use of media increases during childhood, engaging these systems in prevention efforts likely will become more important. Eventually, as the developmentally informative evidence base increases, it will be possible to strategically target multiple levels of processes (e.g., at the individual, family, school, and public policy levels) to reduce the prevalence and severity of underage drinking.

### The Agency Principle

The human organism is an active agent in its own development.

As children mature, they play an increasingly active role in their own development. By the choices they make, the risks they take, the friends they choose, the contexts they select, and the interactions they engage in, children help shape their lives. Development often is accompanied by increasing mobility, choices of friends, access to media, and time unsupervised by adults, which allows young people growing influence over their own experiences. By adolescence, young people play a very active role in decisions that affect their alcohol use and many other risk factors for underage drinking, such as associating with peers who drink, attending parties where there is pressure to drink, joining sport teams that discourage (or encourage) risky behaviors, and watching commercials, films, or other media that glamorize or encourage drinking.

### The Mutually Informative Principle

*Understanding normal or adaptive behavior informs understanding of deviant or maladaptive behavior and vice versa; therefore, it is important to study successful and unsuccessful development, healthy behavior and problem behavior, and pathways to resilience as well as maladaptive outcomes*.

Studies of deviant and normal development are mutually informative. For purposes of underage drinking, the mutually informative principle means that it is important to understand who does not drink as well as who does, pathways to abstinence and appropriate adult drinking as well as pathways to problematic drinking, protective as well as risk factors for underage drinking, the causes underlying drinking cessation and recovery as well as initiation and progression, positive as well as negative effects and outcomes of underage drinking, and normative as well as non-normative drinking.

### The Longitudinal Principle

*Prospective, longitudinal studies are essential for understanding the interplay of the systems that influence development and the many possible pathways toward and away from psychopathology*.

Prospective, longitudinal studies that follow a group of people over time to study outcomes as they unfold are critical to better understanding the processes and pathways toward and away from developmental problems and disorders, including the etiology, initiation, and escalation of underage alcohol use as well as its consequences. Although cross-sectional data are important for descriptive purposes and early stages of developmental understanding, they only represent snapshots of a developmental stream that may mask important turning points as well as individual differences in the processes, timing, and pace of development. In a similar way, retrospective studies that rely on recall of past events may be less informative because they are subject to errors and distortions in human memory.

Longitudinal studies enable the analysis of changes within individuals over time, thereby providing a richer understanding of the antecedents and consequences of alcohol use and AUDs as well as early signs of alcohol problems. Such studies also are useful in determining the effectiveness of interventions and the persistence of their effects over time.

## What’s Next?

This article has provided an overview of a developmental perspective on underage alcohol use. The next three articles examine that perspective in more detail as it applies to underage alcohol use in three age-groups corresponding to childhood, early adolescence, and later adolescence (approximately 0 to 10, 10 to 15, and 16 to 20). Each of these articles is organized with relatively similar content headings, making it possible for readers to follow the flow of development from birth through age 20 by sequentially reading the content found under the same subject headings in each of the three age-group articles. If read in this way, this journal issue provides a sense of the continuous flow of human maturation in a particular area. For example, a reader might be interested in normative development from birth to age 20 or in alcohol-specific risk factors as they unfold over that age span.

On the other hand, the organization of this journal issue by age-group also allows parents, educators, and policymakers to focus on a single age-group and gain an understanding of the multiple interacting developmental processes operating within that group, subject to the caveats raised earlier that children of the same age are not necessarily at the same place on a particular developmental dimension, nor are individual children equally far along in terms of the many different dimensions of human maturation. The final three articles in this journal issue discuss prevention and treatment of alcohol-related problems from a developmental perspective.

## Footnotes

1 For the purpose of this report, underage refers to individuals under the minimum legal drinking age of 21.

2 Effortful control refers to the ability to make oneself perform tedious tasks, such as doing repetitive math homework.
